# COVID‐19 risk in breast cancer patients receiving CDK4/6 inhibitors: literature data and a monocentric experience

**DOI:** 10.1111/tbj.14204

**Published:** 2021-03-06

**Authors:** Maddalena Barba, Eriseld Krasniqi, Laura Pizzuti, Marco Mazzotta, Daniele Marinelli, Greta Giuliano, Francesca Sofia Di Liso, Federico Cappuzzo, Lorenza Landi, Silverio Tomao, Gennaro Ciliberto, Patrizia Vici

**Affiliations:** ^1^ Division of Medical Oncology 2 IRCCS Regina Elena National Cancer Institute Rome Italy; ^2^ Medical Oncology Unit Department of Clinical and Molecular Medicine Sant’Andrea Hospital Sapienza University Rome Italy; ^3^ Department of Radiological, Oncological and Anatomo‐Pathological Sciences, Policlinico Umberto I Sapienza – Università di Roma Rome Italy; ^4^ Scientific Direction IRCCS Regina Elena National Cancer Institute Rome Italy

**Keywords:** CDK4/6 inhibitors, Covid‐19, HR positive/HER2 negative metastatic breast cancer

## Abstract

Substantial changes in the management of cancer patients have been required worldwide in response to the COVID‐19 pandemic. Beyond the due details on the primitive cancer site and setting at diagnosis, these latter adaptions are most commonly exemplified by a significant reduction in the screening of asymptomatic subjects, delays in elective surgery and radiotherapy for primary tumors, and dose reductions and/or delays in systemic therapy administration. Advanced breast cancer patients with hormonal receptor positive, HER2 negative tumors are usually treated with endocrine therapy combined with CDK 4/6 inhibitors as first‐ and second‐line treatment. During the pandemic, experts’ recommendations have suggested the omission or delay of CDK 4/6 inhibitors delivery, or a careful evaluation of their real need due to the hypothesized increased risk of SARS‐Cov‐2 infection and disease possibly related to neutropenia. The inherent literature is sparse and inconsistent. We herein present data on the use of CDK 4/6 inhibitors during the pandemic. The evidence reported punctually reflects the experience matured at our Institution, a comprehensive cancer centre, on the topic of interest.

## 1 INTRODUCTION

Over the past few months, the evidence on severe acute respiratory syndrome coronavirus 2 (SARS‐CoV‐2) infection and inherent disease (Covid‐19) has grown at an impressive rate. Data from the earliest reports on Covid‐19 patients from China conveyed a quite alarming message concerning a higher risk of SARS‐CoV‐2 infection and disease in cancer patients, for whom worse outcomes were described compared to non‐cancer patients.^1^ The limited generalizability of these findings has been amply underlined elsewhere, along with the need of punctual data concerning patient‐, disease‐ and treatment‐related features, which may all plausibly concur to define patients’ immune‐competence and outcome at the time of the of patient‐virus interaction.^2^, ^3^, ^4^, ^5^, ^6^

In cancer patients, the potentially higher risk of SARS‐CoV‐2 infection and disease may be reconciled with the recent administration of cancer‐specific treatments, possibly including surgery, radiotherapy, chemotherapy, and targeted therapies, either singularly or combined. Peculiar pathogenetic aspects have been advocated in support of a separated discussion concerning the risks eventually conferred by immunotherapy.^6^ However, to the best of our knowledge, no questions have been openly and specifically posed, nor evidence‐based opinions expressed, concerning the risk associated with the administration of cyclin‐dependent kinase 4 and 6 (CDK4/6) inhibitors combined with aromatase inhibitors or fulvestrant in hormone receptor positive (HR+), human epidermal growth factor receptor 2 negative (HER2‐) metastatic breast cancer. These latter agents represent the newest frontier of endocrine therapy in HR+/HER2‐ advanced breast cancer. Their widespread use, consistently supported by significantly improved progression‐free survival and overall survival, along with the extremely common hematologic toxicity, justifies our research question.^7^

In brief, palbociclib, ribociclib, and abemaciclib are regulators of cell‐cycle progression, through cyclin D. The interaction of cyclin D with CDK4/6 induces hyperphosphorylation of the retinoblastoma (Rb) gene product, leading to the transition from G1 to S cell‐cycle phase; CDK4/6 inhibitors cause arrest of cell cycle progression. The most common side effects of palbociclib and ribociclib are neutropenia and leucopenia, less frequent observed with abemaciclib. Importantly, neutropenia induced by CDK4/6 inhibitors differs from chemotherapy‐associated neutropenia in several aspects, including grade of toxicity, underlying mechanisms, and time to recovery. In a recent systematic review and meta‐analysis including six eligible randomized trials, the relative risk (RR) for grade ¾ neutropenia was 44.0 (*p* < 0.0001), whereas there was no significant increase in the relative risk of febrile neutropenia compared with endocrine therapy alone (RR: 3.29, *p* 0.06). Indeed, the rate of febrile neutropenia related to CDK4/6 inhibitors is particularly low, about 2–3%, based on data from the registrative trials. Conversely, in first‐line trials of metastatic breast cancer patients treated with citotoxic agents, febrile neutropenia rates raised up to 36%.^8^, ^9^ Importantly, neutropenia induced by CDK4/6 inhibitors differs from chemotherapy‐associated neutropenia in several aspects, including underlying mechanisms, degree of toxicity, and time to recovery. The issues related to the explicative mechanisms and time to recovery are tightly related. Bone marrow suppression from CDK4/6 inhibitors is due to cell‐cycle arrest by decreased hematopoietic stem cells proliferation. This process is rapidly reverted by CDK4/6 inhibitors dose‐reduction or interruption. This makes toxic effects rapidly reversible. Conversely, chemotherapy‐induced neutropenia is caused by apoptotic death of bone marrow progenitor cells, a process which imposes longer time to restoration of the *quo ante* conditions and implies longer time to recovery. In addition, on the long term, due to the lack of DNA damage response following CDK4/6 inhibitors treatment in normal bone marrow–proliferating cells, the risk of secondary hematologic cancers, a known risk of DNA‐damaging chemotherapy, may be lower.^10^, ^11^, ^12^

One of the most debated decisions during the COVID‐19 pandemic relates to the addition of CDK 4/6 inhibitors to endocrine therapy, because of the necessarily more frequent in‐visits of the patients, and because of the immunosuppressive effect. Experts recommendation suggest that, during the pandemic, the decision to add a CDK 4/6 inhibitor to endocrine therapy should take into account the burden of metastatic disease, the sites of disease progression, and to consider the possibility of postpone their use later in the course of the disease.^13^

Though not specifically referred to the patients population and treatment currently debated, evidence on modification of hematological parameters in course of Covid‐19 infection is available. Fan and colleagues presented the outcome of analysis performed in 67 patients admitted to the National Centre for Infectious Diseases (NCID) of Singapore as of February 28, 2020. Patients were all ascertained by RT‐PCR and performed at least one complete blood count (CBC) during their in hospital stay.^14^ In 65 of them with CBC performed on admission, leukopenia was shown in 29.2%. It was usually mild, with only one patient presenting with severe leukopenia. Lymphopenia was observed in 36.9% of these patients, being moderate to severe. Thrombocytopenia, usually mild, was reported in 20% of the patients. These data differ from those reported from China, wherein the lymphopenic patients were 69% and 42% for patients in Wuhan and outside Wuhan, respectively. Patients requiring intensive care unit (ICU) showed a lower absolute lymphocyte count (ALC). No neutropenia was observed, while neutrophilia was commonly reported during the hospitalization, with a median peak of absolute neutrophil count (ANC) of 11,600 in the group of patients requiring ICU, compared with 3,500 in the non ICU subgroup (*p* < 0.001).^14^, ^15^, ^16^

Literature specifically focused on SARS‐CoV‐2 infection and disease in course of the Covid‐19 pandemic is poorly represented when specifically referring to a specific treatment category. On this basis, the evidence from the case‐series from Vuagnat and colleagues appears particularly relevant. These authors described the outcomes of a cohort of 59 breast cancer patients diagnosed with Covid‐19 based on viral RNA testing or radiologic evidence. Among them, 37 (63%) were treated for metastatic breast cancer. Nine patients (24%) from this latter subgroup received CDK4/6 inhibitors. Results from the overall case series of 59 patients were as if follows: 45 (76%) patients recovered completely or were recovering at the time of writing, while 10 (17%) were still followed. Four (7%) patients died from Covid‐19. Significant co‐morbidities were reported for all 4 patients who died. In univariate models, hypertension and age greater than 70 years were the only two factors associated with a higher risk of admission to the ICU and/or death. Treatment with CDK4/6‐inhibitors was not reported among the factors impacting the Covid‐19 disease course.^17^

A report presented during the 2020 AACR Virtual Meeting on COVID‐19 and Cancer suggests that withdrawal or dose‐reduction of CDK 4/6 inhibitors might reduce the risk of infection from COVID‐19 disease. A total of 79 advanced breast cancer patients treated with palbociclib (*n* = 44), ribociclib (*n* = 21), and abemaciclib (*n* = 13) in combination with endocrine therapy were retrospectively recruited and analyzed. At physicians’ discretion regimens were withdrawn or dose adjusted considering age, comorbidities, and previous neutropenia. The analysis was retrospectively performed to evaluate the impact of CDK 4/6 withdrawal/dose adjustment (non‐exposed) on developing COVID‐19 infection. An overall incidence of COVID‐19 of 7.7% was found among all the casistic, 12.8% in exposed patients and 2.7% in non‐exposed patients with a 5.8 odds ratio for exposed patients (*p* 0.077). The incidence of COVID‐19 in patients receiving ribociclib, abemaciclib, and palbociclib was 14.29%, 7.69%, and 4.55%, respectively. Additionally, patients who had a CDK 4/6 inhibitor withdrawal or dose reduction did not show disease progression. The authors conclusions are that, although without statistically significant difference, withdrawn/dose‐reduction of CDK 4/6 inhibitors may reduce the incidence of Covid‐19.^18^

Lastly, it was recently published the first case‐report of an advanced breast cancer patients with COVID‐19 infection while on treatment with a CDK 4/6 inhibitor, namely palbociclib. The patient had prolonged fever, lasting 9 days, dyspnea, weakness and nausea, and palbociclib was held on the first day of her hospital stay. Blood count revealed mild leukopenia and moderate neutropenia; an initial chest x‐ray was with no remarks, and she was tested positive for COVID‐19 (RT‐PCR) from nasopharyngeal swab. Subsequent blood tests showed normalization of the leukopenia/neutropenia and the occurrence of lymphopenia. A new chest x‐ray and a CT scan, performed on day 11, showed bilateral basal infiltrates and multifocal pulmonary peripheral ground glass opacities, with oxygen desaturation on days 12–14 of hospitalization. The authors conclude that the patient experienced an unusually delayed course of COVID‐19 illness, and hypothesize that palbociclib administration prior to the hospital admission caused a short‐term immunosuppressive effect, delaying the classic presentation of the disease.^19^

Concerning the experience matured at our Institution, the IRCCS Regina Elena National Cancer Center is a OECI‐accredited center for cancer care and research, which acted as a Covid‐19‐free cancer hub during the lockdown established in Italy in response to the evolving Covid‐19 pandemic. In agreement with the national policy issued by the Italian Ministry of Health, we adopted restrictive measures mainly based on the postponement of all non‐mandatory institutional accesses. At our Institute, starting from the year 2019, HR+HER2‐advanced breast cancer patients treated with CDK4/6 inhibitors combined with aromatase inhitors or fulvestrant have been invited to participate in a study (Indaco Trial) aimed at the evaluation of biomarkers of efficacy and toxicity, which received formal approval by the Institutional Review Board (IRB) on the January 29, 2019, protocol number: RS1162/18(2179) RC18. At the time of writing, 54 patients have entered this study. The study participants provide a unique occasion to evaluate the following Covid‐19 related outcomes: a. the risk of SARS‐CoV‐2 infection and disease in participants enrolled starting from March 11, 2020, that is, from the date the Covid‐19 was declared a pandemic by the World Health Organization ^20^; b. to provide an estimate of the effects of the lockdown on patients’ accrual at this single study level. Since this trial participants are by eligibility criteria breast cancer patients with an indication to CDK4/6 inhibitors in clinical practice, the comparison between the rate of accrual pre‐ and post‐March 2020 may be intended as a proxy of this specific treatment assignment in course of lockdown. The analysis whose results are reported below were formally approved by the institutional IRB on the July 21, 2020 (protocol number: CE 1388/20).

As concerns the accrual of the patients during the lockdown, among the 54 study participants, 43 were enrolled between January 2019 and the first days of March 2020, while the remaining 11 patients were enrolled between the 09^th^ March, the closest day to the declaration of pandemic, and July 15, 2020. On this basis, the rates of accrual over the about 15 months which preceded the lockdown and the 5 months which followed were 2.86 and 2.2 patients/month, respectively. Thus, no relevant differences emerged.

Focusing on The risk of COVID‐19 infection and disease related to CDK 4/6 administration, none of the 11 patients enrolled following the beginning of the lockdown developed SARS‐Cov‐2 infection and/or disease, neither did the 21 patients who began CDK4/6 administration prior to March 2020 and continued the therapy assigned during the lockdown time‐window. The clinical‐pathological features and treatment administered for patients participating in the previously mentioned trial are shown in Table 1. Data are reported by interval of enrollment, that is, prior to and following the lockdown establishment in Italy.

**TABLE 1:** Descriptive characteristics of the INDACO study participants (*N* = 54)

	Pts enrolled prior to the LD	Pts enrolled following the LD
	*N* (%)	
	43 (70)	11 (20%)
Age (years)		
˂50	19 (44)	5 (45)
≥50	24/56)	6 (54)
Prior neo/adjuvant therapy		
No	12 (28)	8 (73)
Yes	31 (72)	3 (27)
Metastatic at diagnosis		
No	35 (81.4)	6 (54.5)
Yes	8 (18.6)	5 (45.5)
CDK4/6 In combined with		
Aromatase inhibitor	15/35)	1 (9)
Fulvestrant	28 (65)	10 (91)
CDK4/6 In		
Palbociclib	24 (56)	5 (45)
Ribociclib	13 (30)	5 (45)
Abemaciclib	6 (14)	1 (10)

Abbreviations: CDK4/6 In: cycline dependant kinases 4/6 inhibitors; crude number and percentage; INDACO: acronim from the Italian “Valutazione di biomarcatori predittivi di efficacia e tossicità in pazienti affette da carcinoma mammario avanzato HR+HER2‐in trattamento con INibitori Di chinAsi CiclinO‐dipendenti 4/6”; LD lockdown; *N*(%);Pts: patients.

We finally turn back to the original question, that is, “In the Covid‐19 era, is there an increased risk for breast cancer patients treated with palbociclib, ribociclib, or abemaciclib?”. Based on the available literature data and the aforementioned results from our institutional experience, caution is invited in drawing any firm conclusions, mainly due to the still restricted sample size of the patient subsets and considered and the paucity of data from the literature. Further evidence is, thus, warranted to reinforce or disconfirm the findings discussed.

## CONFLICT OF INTEREST

MB, EK, MM, DM, ST, GG, FSDL, FC, LL and GC declare no conflicts of interest. LP received travel grants from Eisai, Roche, Pfizer, Novartis; speaker fees from Roche, Pfizer, Novartis, Gentili. PV received travel grants from Eisai, Roche, Pfizer, Novartis; speaker fees/advisory boards from Roche, Pfizer, Novartis, Gentili, Lilly.

## AUTHOR CONTRIBUTIONS

PV, EK, LP, FC, LL, and MB: conceptualized or designed the study. MM, DM, GG, and FSDL: acquired the data. ST and GC: involved in critical revision. MB, EK, and PV: involved in preparation and final approval of the manuscript.
